# Whole exome sequencing identified mutations of forkhead box I 1 (*FOXI1*), keratin 6 C (*KRT6C*) and gap junction protein delta 2 (*GJD2*) in a low-grade oncocytic tumor of the kidney: a case report

**DOI:** 10.1186/s13000-025-01616-3

**Published:** 2025-02-21

**Authors:** Akinari Kakumoto, Koichi Nishimura, Daisuke Toki, Rika Kasajima, Hajime Kuroda, Yoji Nagashima, Tsunenori Kondo, Yohei Miyagi, Atsuko Masunaga

**Affiliations:** 1 Department of Diagnostic Pathology, Tokyo Women’s Medical University, Adachi Medical Center, Tokyo, Japan; 2 Department of Surgical Pathology, Tokyo Women’s Medical University Hospital, Tokyo, Japan; 3 Department of Urology, Tokyo Women’s Medical University Adachi Medical Center, Tokyo, Japan; 4 Molecular Pathology and Genetics Division, Kanagawa Cancer Center Research Institute, Yokohama, Japan

**Keywords:** Low-grade oncocytic tumor, Kidney, Whole-exome sequencing, FOXI1, Keratin 6c, GJD2, Connexin 36

## Abstract

**Background:**

Low-grade oncocytic tumor (LOT) of the kidney is an emerging entity among renal oncocytic tumors. While the histological features of LOT of the kidney are similar to those of renal oncocytoma, LOT immunohistochemically expresses keratin 7 (KRT7) but not KIT while renal oncocytoma expresses KIT. Molecular analyses of LOTs of the kidney using next generation sequencing revealed those tumors harbor mutations of mTOR-related genes.

**Case presentation:**

An 80-year-old Japanese man with a history of clear cell renal cell carcinoma and prostatic cancer underwent resection of the tumor of the right kidney, 10 mm in diameter, which was monitored for six years. The tumor was histologically composed of oncocytic cells that expressed KRT7, vimentin, SDHA, SDHB and fumarate hydratase, but not KIT, GATA3 and alpha-methylacyl-CoA racemase. We diagnosed the tumor as LOT of the kidney. Whole-exome sequencing of the LOT revealed single nucleotide variants in the DNA-binding region of forkhead box I1 (*FOXI1*), the coil 1B domain of keratin 6 C (*KRT6C*) and the intracytoplasmic region of gap junction delta 2 (*GJD2*), which encodes connexin 36. However, there was no mutations in mTOR-related genes. No copy number alterations were detected in the tumor.

**Conclusions:**

We report three mutations in genes that have not been previously reported in LOT of the kidney. The genes are not related to the mTOR pathway. Therefore, LOT of the kidney might occur through several mechanisms and/or include several types of renal oncocytic tumors.

## Background

Rare oncocytic renal tumors resemble oncocytoma, yet show diffuse keratin 7 (KRT7) staining and negative KIT staining, which would be unexpected for renal oncocytoma (RO) [1]. These tumors led to diagnostic problems for pathologists until 2019, when Trpkov et al. proposed an emerging entity, low-grade oncocytic tumor (LOT) of the kidney, from an analysis of 28 renal oncocytic tumors that immunohistochemically presented KRT7 but not KIT [2]. Prior to the report by Trpkov et al., pathologists had diagnosed oncocytic renal tumors with KRT7 positivity and KIT negativity as an atypical eosinophilic type of chromophobe renal cell carcinoma (eo-ChRCC) or an atypical type of RO [2]. Tumor cells of eo-ChRCC proliferate in a solid pattern, exhibit raisnoid nuclei with irregular contour and are usually positive for KIT [3, 4]. Some authors re-examined oncocytic renal tumors with KRT7 positivity and KIT negativity at their institutions by histological and immunohistochemical analysis and renamed the tumors as LOT of the kidney [5–10]. In 2022, the 5th WHO classification of the renal oncocytic tumors divided renal oncocytic tumors into three groups: RO, chromophobe renal cell carcinoma (ChRCC) and other oncocytic tumors, which include LOT of the kidney [11]. As described in previous reports and the WHO classification, the histological features of LOT include oncocytic tumor cells that present a solid growth pattern and round nuclei of tumor cells. Thus, the histological features of LOT are similar to those of RO rather than eo-ChRCC. An immunohistochemical feature of LOT is that the tumor expresses KRT7 but not KIT [11]. Thus, LOT of the kidney can be diagnosed the using histological and immunohistochemical studies in routine medical practice.

Several reports have provided the molecular information of LOT of the kidney. The reports revealed chromosomal anomalies [2, 5] and impairment of the mammalian target of rapamycin (mTOR) pathway in tumors by panel or targeted next-generation sequencing (NGS) [6, 7, 12–15]. Using whole exome sequencing (WES) and Sanger sequencing of five LOTs, Kapur et al. found impairments of the mTOR pathway in five LOTs analyzed in the study [8]. Thus, impairment of the mTOR pathway is thought to be a mechanism of tumorigenesis of LOT of the kidney.

We experienced a patient with LOT of the kidney and subjected the tumor to WES analysis in order to determine whether there may be molecular anomalies of the LOT of the kidney other than impairment of mTOR-pathway.

## Case presentation

An 80-year-old Japanese man with a history of clear cell renal cell carcinoma and prostatic cancer underwent robotic partial nephrectomy and adrenalectomy for the tumors of the right kidney and right adrenal gland. The tumor of the right kidney, 10 mm in diameter, had been monitored for six years.

**Fig. 1:**
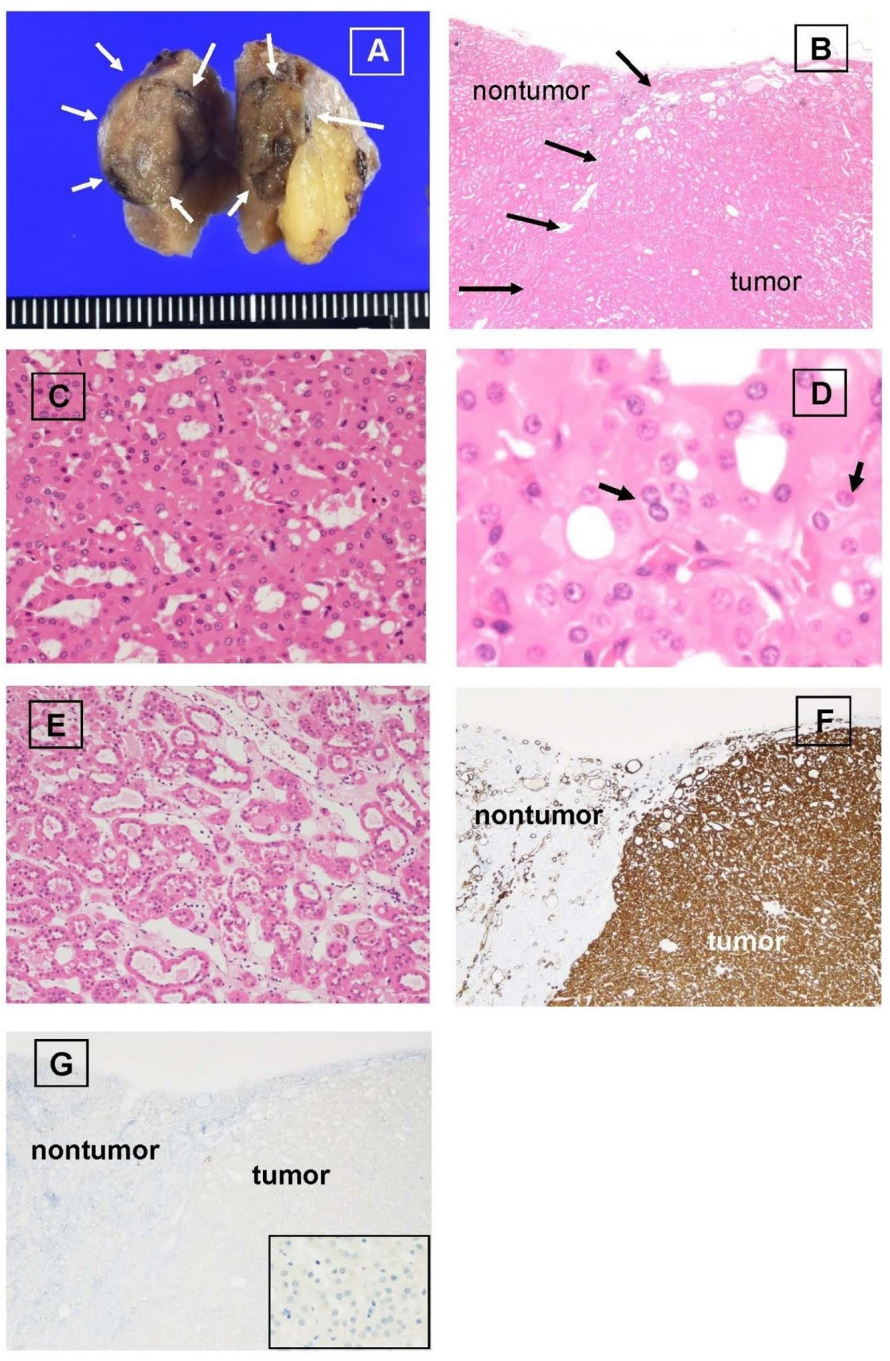
Macroscopic findings and histological and immunohistochemical findings of the low-grade oncocytic tumor: **A**: Macroscopic appearance of the resected tumor of the right kidney (partial nephrectomy). The tumor, 10 mm in diameter, was located beneath the renal capsule and is indicated with arrows. The tumor was solid and tan. **B**: Hematoxylin and eosin (HE)-stained section showing the boundary (arrows) between the tumor and the nontumorous kidney. There was no fibrous capsule at the boundary. The tumor cells showed solid growth pattern. (Original magnification x20). **C**: Microscopic appearance of the tumor. The tumor was composed of oncocytic tumor cells. The tumor cells formed tight trabecular or small glands in the area showing solid growth patten. (HE section, original magnification x200). **D**: Higher magnification image of the tumor. Tumor cells exhibited round nuclei. Some cells show a few inconspicuous nucleoli. A few tumor cells showed a vague perinuclear halo (arrows). (HE section, original magnification x600). **E**: Edematous area of the tumor. The area was located in the center of the tumor. The tumor cells formed irregular glands. Some tumor cells were scattered individually, and myoepithelial cell–like cells with small nuclei and scant cytoplasm were scattered in the stroma. (HE section, original magnification x100). **F**: Immunohistochemical staining of KRT7. All tumor cells strongly expressed KRT7. (Original magnification x20). **G**: Immunohistochemical staining of KIT. The tumor cells were diffusely negative for KIT (original magnification x20). Inset image shows KIT-negative tumor cells (original magnification x200)

The tumor of the right kidney, 10 mm in diameter, was macroscopically located in the renal cortex and pushed against the renal capsule; it was well-demarcated. The cut surface of the tumor was brown without capsule or necrosis (Fig. 1A). The tumor lacked a surrounding fibrous capsule separating it from the surrounding nontumorous kidney (Fig. 1B). There was no central scar. The tumor cells were oncocytic and proliferated in a tubular to nested pattern (Fig. 1C). No vacuolated or pale tumor cells were detected. The nuclei were uniformly round without irregular contours and a few nucleoli were occasionally detected. Some tumor cells had a vague perinuclear halo, and a few tumor cells had two nuclei (Fig. 1D). No mitotic figure was observed in the hematoxylin and eosin–stained slide. Some parts of the tumor showed edematous areas, in which some tumor cells formed dilated tubules or some tumor cells were scattered throughout (Fig. 1E). Immunohistochemical staining revealed diffuse KRT7 (Fig. 1F), vimentin, PAX8, e-cadherin, SDHA, SDHB and fumarate hydratase expression in tumor cells. No KIT (Fig. 1G), CD10, CA9, GATA3 or alpha-methylacyl-CoA racemase was detected in the tumor cells. The histological and immunohistochemical findings were consistent with the features of LOT of the kidney.

**Fig. 2:**
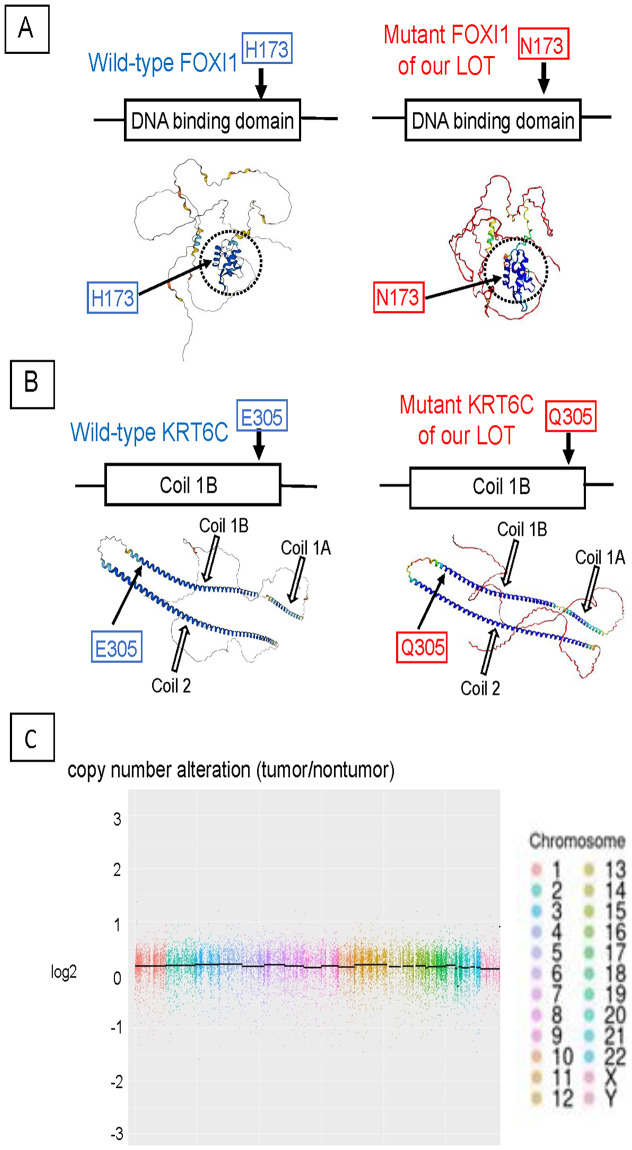
Somatic mutations and copy numbers of the low-grade oncocytic tumor (LOT). **A**: The wild-type forkhead box I 1 (FOXI1) protein and the predicted mutant FOXI1 harboring the mutation identified in the LOT in this study. Three-dimensional structures shown on bottom were drawn by AlphaFold2. The circle (dotted line) indicates the DNA binding domain, which was drawn as a highly confident area by AlphaFold2 (blue ribbons). The DNA binding region of the mutant FOXI1 showed a similar structure as that of wild-type FOXI1. **B**: Wild-type keratin 6c (KRT6C) protein and the predicted mutant KRT6C in the LOT. Three-dimensional structures shown on bottom were drawn by AlphaFold2. Three coils, coli 1 A, coil 1B and coil 2, of KRT6C were drawn as a highly confident area by AlphaFold2 (blue ribbons). The location and structure of the three coils in the mutant KRT6C were similar to those of wild-type KRT6C. **C**: Copy number alteration in the LOT compared with the nontumor kidney surrounding the LOT. Black bar in the figure indicates the mean copy number of each chromosome. No copy number alteration was detected on each chromosome

To examine the molecular anomalies of the LOT, we performed WES with matched tumor and nontumor samples collected from formalin-fixed, paraffin-embedded sections using laser capture microdissection. We estimated the proportion of neoplastic cells in the tumor area to be 80%. Total DNA was extracted from samples using the GeneRead DNA FFPE kit (Qiagen, Hilden, Germany). Exome capture was performed using Agilent SureSelect Human All Exon V6 (Agilent Technologies Inc., Santa Clara, CA, USA). WES was performed with the Illumina NovaSeq6000 (Illumina Inc. San Diego, CA, USA). The quality of each sample was checked with FastQC version 0.11.9 (https://www.bioinformatics.babrah.ac.jp). The number of original pair end reads of the tumor sample were 54,087,625 and the number in the nontumor sample were 48,905,509. The mean read coverage of the tumor sample was 123x and that of the nontumor sample was 184x. We aligned and annotated the sequence data to the human reference genome (GRCh38/hg38) using Illumina/Strelka v2.9.10, Illumina/Manta v1.6.0 and SnpEff. To detect tumor-specific mutations, we filtered the data using the following criteria: (1) selecting “Pass” by VCF (variant call format: https://samtools.github.io/hts-specs/VCFv4.3) to exclude mis-calling, (2) selecting “High” or “Moderate” using SnpEff, (3) excluding Japanese SNP using dbSNP (NCBI https://www.ncbi.nlm.gov) and Human Genetic Variation Database (https://www.hgvd.genome.med.kyoto-u.ac.jp), (4) excluding “benign” or “likely benign ” variations on ClinVar (https://www.ncbi.nlm.nih.gov/clinvar/), (5) read depth **≥** 50x both in tumor and nontumor samples, (6) no variant allele frequency (VAF) in nontumor and > 10%VAF in tumor, and (7) prediction of detrimental effects for nonsynonymous mutation that Sorting Intolerant From Tolerant (SIFT, https://sift.bii.a-star.edu.sg), Functional Analysis through Hidden Markov Models (FATHMM, fathmm.biocompute.org, uk), PROVEAN (https://www.jcvi.org/research/provean) and MetaSVM (https://github.com/bioinform/metasv/archive/0.5.2.tar.gz) had all estimated as “damaging.” We identified single nucleotide variants (SNVs) in the coding regions of forkhead box I 1 (*FOXI1*), keratin 6C (*KRT6C*) and gap junction protein delta 2 (*GJD2*). The SNV c.517C > A of *FOXI1* results in p. His173Asn, which is located in the DNA-binding domain of FOXI1. The SNV c.913G > C of *KRT6C* results in p. Glu305Gln, which is located in the coil 1B region of the intermediate filament. The SNV c.517G > T of *GJD2* results in p. Glu173*, which is located in the cytoplasmic loop of connexin 36, which is encoded by *GJD2* (Table 1, Fig. 2).

**Table 1:** Mutations in the low-grade oncocytic tumor (LOT)

position	gene	mutation of LOT	VAF of LOT	read depth LOT/Nontumor	predicted change of protein	annotation Impact	SIFT prediction	FATHMM prediction	PROVEAN prediction	MetaSVM prediction
chr5: 17,010,647	*FOXI1*	c. 517 C > A	43.4	366/324	p.H173N	Moderate	D	D	D	D
ch12: 52,471,296	*KRT6C*	c. 913 G > C	38.6	498/420	p.E305Q	Moderate	D	D	D	D
chr15: 34,752,927	*GJD2*	c.517 G > T	28.3	187/164	p.E173*	High				

D: Damaging; FOXI1: forkhead box I 1; GJD2: gap junction protein delta 2; KRT6C: keratin 6 C; LOT: low-grade oncocytic tumor; VAF: variant allele frequency

To investigate whether the nonsynonymous mutations of *FOXI1* and *KRT6C* influence the three-dimensional structure of the protein domains, we used ColabFold-AlphaFold2 (https://colab.research.google.com/github/sokrypton/ColabFold/blob/main/AlphaFold2.ipynb) to analyze the mutant protein structures. While SIFT, FATHMM, PROVEAN and MetaSVM had all predicted that the nonsynonymous mutations would cause detrimental effects on both FOXI1 and KRT6C protein structures, analysis of the structures predicted by AlphFold2 indicated that the DNA-binding domain of the mutant FOXI1 protein and the coil 1B domain of the mutant KRT6C protein were similar to the respective domains in the wild-type proteins (Fig. 2A and B). In contrast, the mutation in *GJD2* identified in the LOT sample led to a truncation of connexin 36, which likely impacts the function of connexin 36.

We further analyzed the mutations to determine whether mTOR pathway–related genes were mutated in the LOT. We filtered the data following the laxer criteria as follows: (1) selecting “Pass” by VCF, (2) selecting “High” or “Moderate” using SnpEff, (3) excluding Japanese SNP using dbSNP and Human Genetic Variation Database, (4) read depth **≥** 5x both in tumor and nontumor samples, (6) no VAF in nontumor and > 5% VAF in tumor and (7) prediction of detrimental effects for nonsynonymous mutations that one or more of SIFT, FATHMM, PROVEAN and MetaSVM had estimated as “damaging.” After filtering, the results identified 62 mutations in the LOT that were predicted to lead to detrimental effects. Among the 62 mutated genes, there were no mTOR pathway–related genes.

We then analyzed copy number variations in the LOT compared with the nontumor kidney tissue surrounding the tumor using DNA Copy version 1.56.0 (https://bioconductor.org/packages/release/bioc/html/DNAcopy.html). We detected no alteration of copy numbers in the LOT (Fig. 2C).

The tumor of the right adrenal gland was a metastatic lesion derived from the clear cell renal cell carcinoma of the left kidney. Six years prior, the patient had undergone laparoscopic partial nephrectomy for clear cell renal cell carcinoma of his left kidney, pT3a. Four years ago, he underwent total prostatectomy because of prostatic acinar adenocarcinoma, pT2. He had no family history of cancer. During periodic clinical follow-up, the patient did not show recurrence or distant metastasis for 24 months after resection of both the LOT and metastatic carcinoma of clear cell renal cell carcinoma. He did not receive chemotherapy or molecular targeted drugs for the clear cell renal cell carcinoma and prostatic cancer before resection of the metastatic renal cell carcinoma in the right adrenal gland and the LOT of the right kidney.

## Discussion and conclusions

The definition of LOT of the kidney has been established. Those tumors are histologically similar to RO, but immunohistochemically express KRT7 but not KIT. The LOT in the current patient was histologically similar to RO and exhibited KRT7 but not KIT expression. Several reports using panel/targeted NGS or WES to analyze LOTs of the kidney revealed mutations of mTOR pathway–related genes [6–8, 12–15]. Impairment of the mTOR-pathway is thought to be a mechanism of tumorigenesis of LOT of the kidney. We analyzed our tumor using WES to determine whether the tumor also showed alteration of mTOR-related genes or the other gene mutations, which might be another mechanism of tumorigenesis of LOT of the kidney.

Our results of WES on the LOT revealed mutations in three genes: *FOXI1*, *KRT6C* and *GJD2*. Mutations of these genes, which are unrelated to mTOR pathway, have never been reported in LOTs of the kidney. Kapur et al. analyzed five LOTs using WES and found mutations of mTOR-related genes but not the three mutations detected in our study.

A study in mice found that FOXI1 was a master regulator of proton pump subunits [16] and that intercalated cells (ICs), located in the collecting duct of the kidney, expressed higher *Foxi1* mRNA levels than non-ICs [17]. RO is considered to originate from ICs [1]. Molnar et al., Tong et al. and Skala et al. reported that most of the examined RO tumors in their studies immunohistochemically expressed FOXI1 [18–20], while Morini et al. and Chen et al. reported that LOTs, which showed mutations of mTOR-related genes on panel NGS in their study, were immunohistochemically negative for FOXI1 [6, 13]. In *Tsc1* knockout mice, deletion of FOXI1 completely inhibited mTORC1 activation [21]. Therefore, the LOT in the current study with *FOXI1* mutation and no mutations of mTOR-related genes still may have impairment of the mTOR pathway in the tumor cells. We analyzed the current tumor using WES and did not examine noncoding regions including expression-regulatory factors for mTOR-related genes. Thus, we cannot rule out the possibility that the current LOT had no functional TSC protein. In the future, whole genome sequencing should be performed to accurately determine the molecular alterations of LOT of the kidney.

Keratin intermediate filaments are formed by heterodimers of type I and type II keratins. The two keratin chains form a parallel coiled coil [22]. The three-dimensional structure of the predicted mutant KRT6C protein containing the *KRT6C* mutation identified in the current LOT was similar to that of wild-type KRT6C protein, suggesting that the predicted protein may still be able to form a coiled coil with type I keratin. Whether there are KRT6C-expressing cells in the kidney is unknown. Furthermore, whether KRT6C plays a role in cancer has not been completely clarified. However, a lung adenocarcinoma with the same *KRT6C* mutation detected in the current patient is registered in the Catalog of Somatic Mutations in Cancer database (COSMIC, https://cancer.sanger.ac.uk/cosmic). Hu et al. reported that lung adenocarcinoma with high expression of *KRT6C* mRNA showed poor prognosis and lung adenocarcinoma cells with small interfering RNA–mediated downregulation of *KRT6C* mRNA showed reduced migration compared with control cells [23]. Most patients with LOT show a good prognosis [2, 5, 6, 8–10], and the LOT in the current patient had not enlarged over the 6 years of monitoring. Furthermore, no recurrence or metastasis was observed during the 24 months after resection. Whether mutation of the *KRT6C* gene in the current tumor is related to clinical aspect of the patient is unclear.

*GJD2* encodes connexin 36, which is located on the cellular membrane. A previous study reported that connexin 36 was related to Mg^2+^ ion permeability [24]. In animal kidney, ICs transport Ca^2+^ ions through the cytoplasm, while Mg^2+^ is transported by cells of distal convoluted tubules [25]. Whether connexin 36 plays roles in the adult human kidney remains unknown. Notably, the *GJD2* mutation observed in the current tumor may lead to severe disruptions in connexin 36 structure and compromise or inhibit the function of the protein.

In conclusion, here we present a patient with LOT of the kidney and report three somatic mutations that have not been reported in LOTs of the kidney. The tumor did not show mutations of mTOR-related genes that were previously reported by several authors who analyzed molecular alterations using NGS. However, a few LOTs showed no mutation of mTOR-related genes using panel/targeted NGS [7, 14]. Our results suggest that LOTof the kidney may occur through several mechanisms and indicate the possibility that LOT of the kidney might include several types of renal oncocytic tumors.

## Data Availability

The datasets supporting the conclusions of this article are included within the article.

## Abbreviations

RO: Renal oncocytoma
LOT: Low-grade oncocytic tumor
eo-ChRCC: Eosinophilic type of chromophobe renal cell carcinoma
mTOR: Mammalian target of rapamycin
WES: Whole exome sequencing
VAF: Variant allele frequency
FOXI1: Forkhead box I 1
KRT6C: Keratin 6c
GJD2: Gap junction protein delta 2
IC: Intercalated cell
